# MCDA or preference-based social welfare functions?

**DOI:** 10.1186/s12962-018-0122-y

**Published:** 2018-11-09

**Authors:** J. P. Sevilla

**Affiliations:** 1 Data for Decisions, LLC, Waltham, MA USA; 2 Harvard Chan School of Public Health, Boston, MA USA

**Keywords:** Social welfare functions, Multicriteria decision analysis, Health, Wealth, Well-being

## Abstract

Preference-based social welfare functions (pbSWF) perform better at reconciling competing personal and social goals than typical forms of MCDA. Its virtues are (a) its respect for people’s own judgments about the relative values of health, wealth, and other broad benefits within their lives (non-paternalism) and (b) its conformity with reasonable ethical axioms. Any discrepancy between an MCDA objective function and that implied by pbSWF suggests the former’s failure to respect non-paternalism and reasonable ethical principles. The pbSWF approach is implementable using micro-econometric evidence on personal preferences over health, wealth, and other broad benefits; and surveys of the general population or their representatives to ascertain the social acceptability of certain ethical axioms and the degree of inequality aversion.

## Background

I challenge MCDA exponents to prove their approach superior to using preference-based social welfare functions (pbSWF) to reconcile competing personal and social goals. pbSWFs were introduced and developed by Bergson [1] and Samuelson [2]. Recent expositions and defenses of the SWF approach include Adler [3] and various chapters in Adler and Fleurbaey [4].

The pbSWF approach uses (1) personal preferences to aggregate up the various broad benefits of health into a scalar index of over-all personal well-being *x*, and (2) reasonable normative axioms to determine the functional form of society’s objective function linking the twin social goals of efficiency and equity with respect to *x*. The pbSWF approach’s virtues are its respect for (a) people’s own judgments about the relative values of health, wealth, and other broad benefits within their lives (non-paternalism) and (b) the reasonable ethical principles underlying the axioms. Any discrepancy between an MCDA objective function and that implied by pbSWF suggests the former’s failure to respect non-paternalism and reasonable ethical principles.

The economic literature shows how to use utility theory and quantitative data from the general population to parametrize and calibrate a measure of lifetime personal utility as a function of broad benefits such as health, wealth, and risk protection (see e.g. Murphy and Topel [5]). This utility which we can denote by *x* reflects, and therefore respects, the trade-offs people make among these goods within their own lives.

Now impose some classical ethical axiomatic restrictions on how society pursues efficiency and equity with respect to well-being *x* (see Moulin [6]): (i) strong Pareto (all else equal, improving any person’s well-being raises social welfare), (ii) impartiality (the value of social welfare depends only on which levels of well-being obtain and not on who enjoys which levels), (iii) continuity (a technical condition I ignore for simplicity), (iv) independence of unconcerned individuals (i.e. the choice among policies does not depend on the well-being of those equally well-off under the policies), (v) independence of common scale (multiplying everyone’s well-being by the same constant does not affect policy choice), and (vi) Dalton’s principle of transfers (all else equal, a non-leaky transfer Δ*x* from a better-off to worse-off person, if small enough not to make the latter better off than the former, raises social welfare).

These axioms constrain the SWF to have one of three forms:

1 $${W = \sum \limits_{i = 1}^{N} x_{i}^{p} \quad for\; 0 < p < 1}$$

2 $${W = \sum \limits_{i = 1}^{N} \ln x_{i} \quad for\;p = 0}$$

3 $${W = \sum \limits_{i = 1}^{N} - x_{i}^{p} \quad for\;p < 0}$$

Here, *i* represents an individual, *N* is the number of individuals in society, and *p* measures society’s aversion to inequality in over-all personal well-being *x*. As *p* → 1 this aversion disappears and *W* approaches utilitarianism, lower values of *p* involve greater inequality aversion, and *p* → − ∞ leads to a Rawlsian Leximin SWF reflecting concern for only the worst off.

Any SWF satisfying (i)–(iii) and (vi) can be rewritten as (See Deaton [7]):

4 $$\begin{array}{*{20}c} {W = \bar{x}\left( {1 - I} \right)} \\ \end{array}$$

where $\bar{x}$ is average well-being, and *I* is some inequality measure. For example, the inequality measure corresponding to the SWF in (1) is:

5 $$I = 1 - \left( {\frac{1}{n}\sum\limits_{{i = 1}}^{N} {\left( {\frac{{x_{i} }}{{\bar{x}}}} \right)^{p} } } \right)^{{\frac{1}{p}}} \quad for\;0 < p < 1,$$

Reasonable ethical axioms, therefore, tightly constrain the functional form linking efficiency and equity measures, and the functional form of the equity measure.

Many MCDA analyses in this conference and the literature purport to encompass the criteria of efficiency and equity. Yet they have objective functions departing significantly from (4) and expressions like (5), which suggests they violate reasonable ethical axioms and produce rankings with questionable ethical justification. These analyses also aggregate broad benefits without relying on preference information, suggesting they fail to respect people’s autonomous judgments about their own well-being.

The pbSWF approach, which respects both preferences and reasonable ethical axioms, is currently unused in real-world policy evaluation settings, though different pieces of it have been implemented in various economic and policy literatures. For example, the literature on the Value of a Statistical Life and the Lifecycle Model show how to use preference information to aggregate health, wealth, and other broad benefits into a measure of over-all personal well-being. See e.g. Murphy and Topel [5]. The literature on inequality measurements using Gini coefficients is founded on the same family of theoretical work as pbSWFs. See e.g. Deaton [7]. There is a growing literature on using surveys to elicit people’s preferences regarding fairness and distribution. See e.g. Robson et al. [8]. Thus, making progress on the preference-based SWF approach largely requires combining different streams of existing work rather than starting from scratch.

The pbSWF approach is wholly implementable. It would use micro-econometric evidence on personal preferences in the general population to aggregate broad benefits into a measure of personal well-being (See e.g. Murphy and Topel [5]). It would rely on consultations and surveys of the general population or their representatives to ascertain social acceptability of axioms (i)–(vi) and the degree of inequality aversion.

## Conclusion

The pbSWF approach compares favorably on non-paternalistic and ethical axiomatic grounds with typical forms of MCDA. Given the feasibility of its implementation, the research and policy communities should more fully investigate its potential to guide real-world policy choices in settings where multiple criteria such as health, well-being, and equity matter.
